# Usefulness of bilateral traction method for endoscopic submucosal dissection of superficial pharyngeal cancer

**DOI:** 10.1055/a-2638-3159

**Published:** 2025-07-17

**Authors:** Ryosuke Ikeda, Hiroaki Kaneko, Hiroki Sato, Kohei Yoshida, Nobuhiko Oridate, Shin Maeda

**Affiliations:** 1 Department of Gastroenterology, Yokohama City University Graduate School of Medicine, Yokohama, Kanagawa, Japan; 2 Department of Otolaryngology, Yokohama City University Graduate School of Medicine, Yokohama, Kanagawa, Japan

Pharyngeal endoscopic submucosal dissection (ESD) has been increasingly performed as a minimally invasive treatment, similar to gastrointestinal ESD 1 2 . However, the hypopharynx presents a challenging treatment area due to its complex anatomical structure and interference from the intubation tube, which limits the available working space. Various traction methods have been proposed to address these challenges 3 4 . Previously, we reported the efficacy of the bilateral traction (BLT) method for colorectal ESD 5 . Herein, we report a case in which BLT facilitated hypopharyngeal ESD ( Video 1 ).

Bilateral traction method in pharyngeal endoscopic submucosal dissection.Video 1

A 62-year-old man with reflux esophagitis underwent esophagogastroduodenoscopy, which revealed a reddish, protruding lesion in the right arytenoid ( Fig. 1 ). Biopsy results confirmed squamous cell carcinoma. The patient was referred to our hospital for pharyngeal ESD. Under general anesthesia, an otolaryngologist inserted a curved rigid laryngoscope (Nagashima Medical Instruments, Tokyo, Japan), and a mucosal incision was initiated using a dual knife (Olympus Medical Systems Co., Tokyo, Japan). However, the limited working space around the larynx made this procedure challenging ( Fig. 2 ). After completing the circumferential incision, subepithelial dissection was initiated; however, dissection around the larynx remained difficult. Therefore, we attached an EZ clip (HX-610-090; Olympus Medical Systems Co., Tokyo, Japan) with a double thread to the specimen for traction ( Fig. 3 ). Each segment of the doubled thread was pulled in the left and right directions to improve the field of view for dissection. Initially, we dissected the pyriform sinus side by pulling the left thread ( Fig. 4 ), which reduced interference from the intubation tube and allowed for sufficient dissection. The right thread was subsequently pulled to facilitate dissection of the central side of the arytenoid ( Fig. 5 ). Subsequently, en bloc resection was achieved with negative margins.

**Fig. 1:**
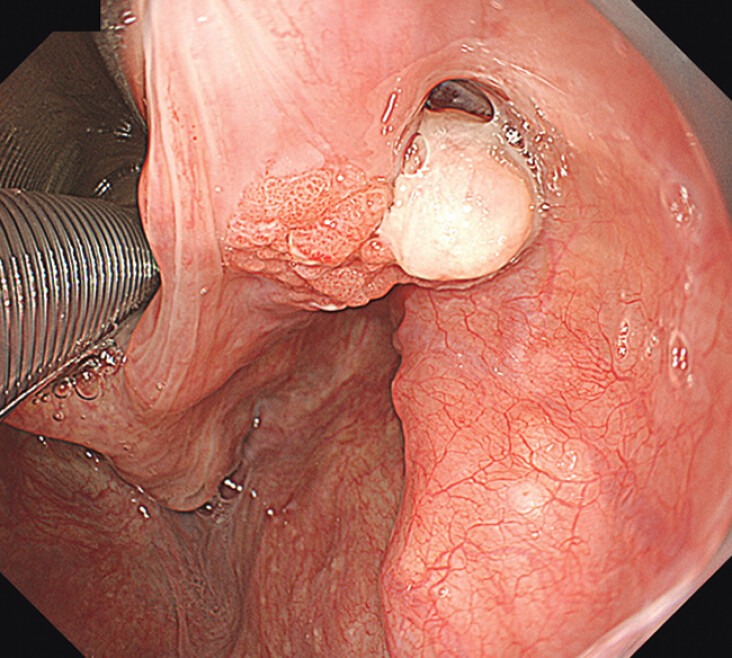
White-light imaging reveals a reddish protruded lesion in the right arytenoid.

**Fig. 2:**
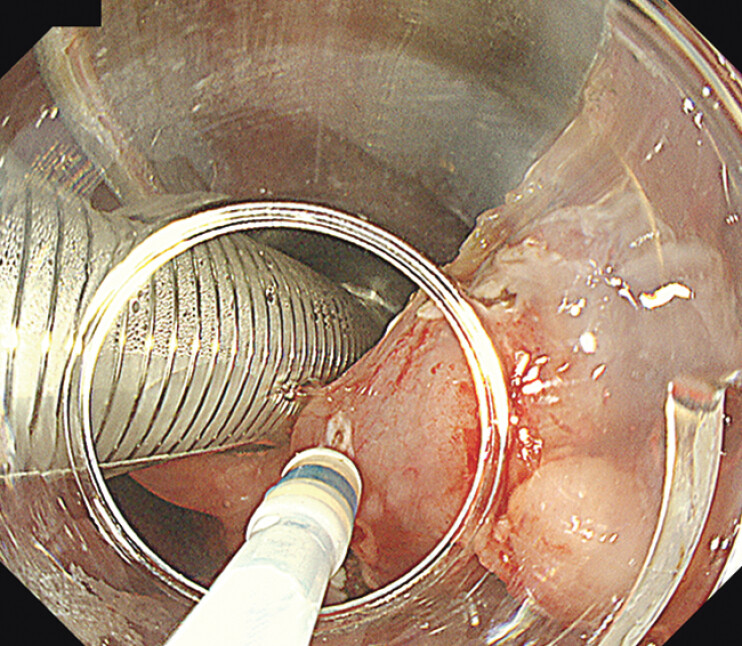
The narrow working space and interference with the intubation tube around the larynx make treatment difficult.

**Fig. 3:**
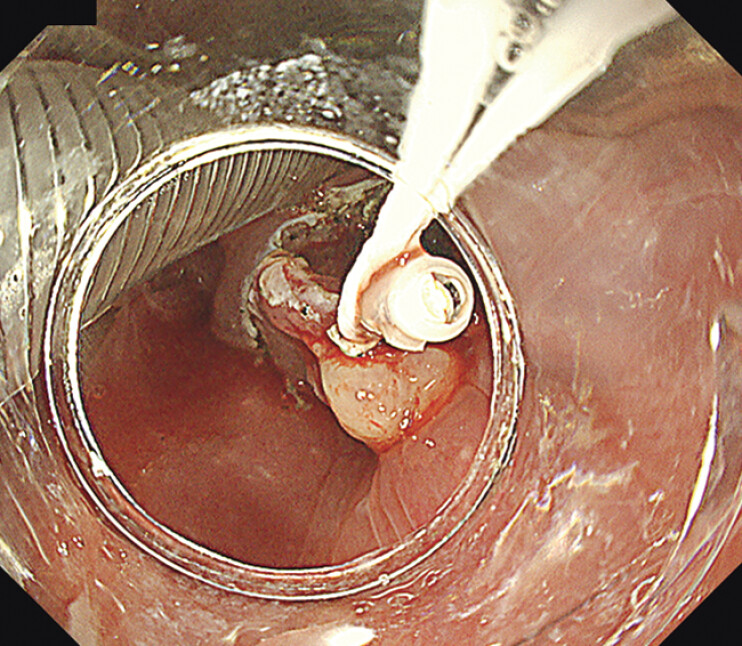
Traction is performed using a clip with a thread. Each thread can be pulled in the left and right directions.

**Fig. 4:**
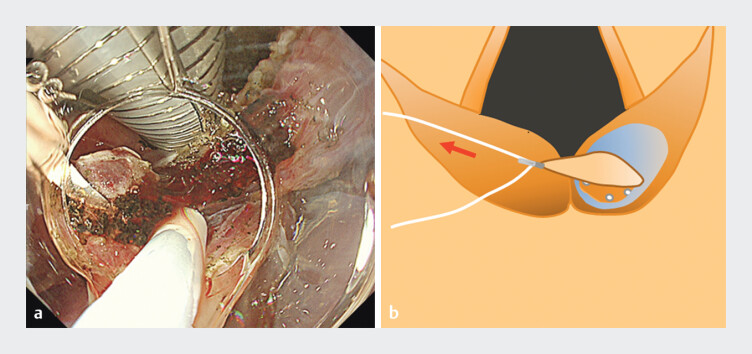
**a** Dissection of the pyriform sinus side. Countertraction to the left makes it easier to maintain the resection view and reduces interference with the intubation tube. **b** Schema of ( **a** ). The working space for dissection around the larynx is maintained by pulling the left thread.

**Fig. 5:**
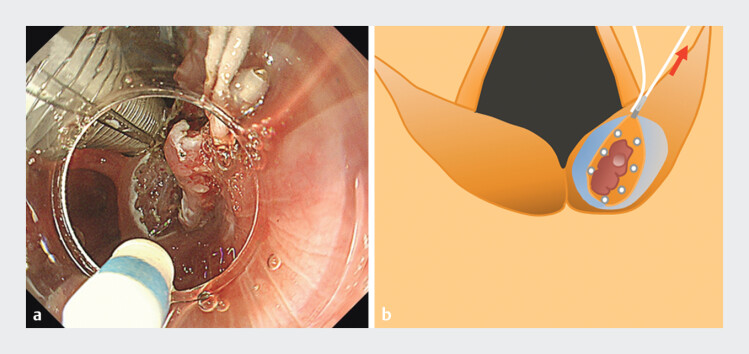
**a** Dissection of the central side of the arytenoid. Countertraction to the right makes it easier to maintain the resection view and keep a distance from the intubation tube. **b** Schema of ( **a** ). The working space for dissection around the larynx is maintained by pulling the right thread.

The BLT method provided adjustable visualization for the endoscopist, which was useful in the hypopharynx, where the working space is narrow.

Endoscopy_UCTN_Code_TTT_1AO_2AG_3AD

## Notes

Endoscopy E-Videos https://eref.thieme.de/e-videos
*E-Videos* is an open access online section of the journal *Endoscopy* , reporting on interesting cases and new techniques in gastroenterological endoscopy. All papers include a high-quality video and are published with a Creative Commons CC-BY license. Endoscopy E-Videos qualify for HINARI discounts and waivers and eligibility is automatically checked during the submission process. We grant 100% waivers to articles whose corresponding authors are based in Group A countries and 50% waivers to those who are based in Group B countries as classified by Research4Life (see: https://www.research4life.org/access/eligibility/ ). This section has its own submission website at https://mc.manuscriptcentral.com/e-videos .

## References

[1] Kuwabara T Hiyama T Oka S Clinical features of pharyngeal intraepithelial neoplasias and outcomes of treatment by endoscopic submucosal dissection Gastrointest Endosc 2012 76 1095 1103 10.1016/j.gie.2012.07.032 23022050

[2] Hanaoka N Ishihara R Takeuchi Y Endoscopic submucosal dissection as minimally invasive treatment for superficial pharyngeal cancer: a phase II study (with video) Gastrointest Endosc 2015 82 1002 1008 10.1016/j.gie.2015.06.021 26234696

[3] Iizuka T Kikuchi D Hoteya S A new technique for pharyngeal endoscopic submucosal dissection: peroral countertraction (with video) Gastrointest Endosc 2012 76 1034 1038 10.1016/j.gie.2012.07.013 22906853

[4] Minami H Tabuchi M Matsushima K Endoscopic submucosal dissection of the pharyngeal region using anchored hemoclip with surgical thread: A novel method Endosc Int Open 2016 4 E828 E831 10.1055/s-0042-108802 27540568 PMC4988839

[5] Ikeda R Kaneko H Sato H Bilateral traction method using a clip with thread for rectal endoscopic submucosal dissection Endoscopy 2024 56 E1131 E1132 10.1055/a-2496-2899 39689895 PMC11652080

